# Downhill hiking improves low-grade inflammation, triglycerides, body weight and glucose tolerance

**DOI:** 10.1038/s41598-021-93879-1

**Published:** 2021-07-15

**Authors:** Heinz Drexel, Arthur Mader, Christoph H. Saely, Gerda Tautermann, Jörn F. Dopheide, Alexander Vonbank

**Affiliations:** 1 Vorarlberg Institute for Vascular Investigation and Treatment (VIVIT), Feldkirch, Austria; 2 Division of Angiology, Department of Internal Medicine, Cantonal Hospital Graubuenden, Chur, Switzerland; 3 Private University of the Principality of Liechtenstein, Triesen, Liechtenstein; 4 Drexel University College of Medicine, Philadelphia, PA USA; 5 Department of Medicine I, Academic Teaching Hospital Feldkirch, Feldkirch, Austria; 6 Department of Medicine, Academic Teaching Hospital Bregenz, Bregenz, Austria; 7 VIVIT Institute, Academic Teaching Hospital Feldkirch, Carinagasse 47, 6807 Feldkirch, Austria

**Keywords:** Dyslipidaemias, Metabolism, Fat metabolism, Metabolic diseases, Diabetes, Dyslipidaemias, Lifestyle modification, Preventive medicine, Patient education

## Abstract

Exercise is a well-established tool for cardiovascular risk reduction. Particularly eccentric exercise, which essentially means walking downwards could favour more people becoming physically active. With the present controlled study, we tested the hypothesis that eccentric exercise can improve insulin sensitivity, triglyceride handling, body mass index, glucose tolerance and inflammation. We allocated 127 healthy sedentary individuals to one of two groups: (i) an active group of 102 individuals walking downwards a predefined route three to five times per week over two months, covering a difference in altitude of 540 m; for the upward route a cable car was used, for which adherence was recorded electronically and (ii) a matched control group of 25 individuals who stayed sedentary. Fasting and postprandial metabolic profiles were obtained at baseline and after two months. Compared to baseline, eccentric exercise significantly improved HOMA insulin resistance (1.94 ± 1.65 vs. 1.71 ± 1.36 (µU^−1^ ml) × ((mmol/l)^−1^22.5); p = 0.038) and resulted in a decrease in fasting glucose (97 ± 15 vs. 94 ± 9 mg dl^−1^; p = 0.025) and glucose tolerance (238 ± 50 vs. 217 ± 47 mg dl^−1^ h^−1^; p < 0.001), whereas these parameters did not change significantly in the control group. Eccentric exercise significantly improved triglyceride tolerance (1923 ± 1295 vs. 1670 ± 1085 mg dl^−1^ h^−1^; p = 0.003), whereas triglyceride tolerance remained unchanged in the control group (p = 0.819). Furthermore, body mass index (27.7 ± 4.3 vs. 27.4 ± 4.3 kg m^−2^; p = 0.003) and C-reactive protein (0.27 ± 0.42 vs. 0.23 ± 0.25 mg dl^−1^; p = 0.031) were significantly lowered in the eccentric exercise group but not in the control group. Downhill walking, a type of exercise is a promising unusual exercise modality with favorable effects on body mass index, insulin action, on postprandial glucose and triglyceride handling and on C-reactive protein.

**ClinicalTrials.gov Identifier**: NCT00386854.

## Introduction

Among lifestyle interventions, exercise is a potent tool for cardiovascular risk reduction. Over decades, evidence has accumulated that exercise improves insulin sensitivity, glucose tolerance, triglyceride clearance from blood, and reduces fat mass, subclinical inflammation and body weight^1, 2^. Research provides evidence for the effectivity of physical activity in primary prevention but its implementation into practice is often not successful^3^ leading to a “research-to-practice gap”^4^. Individuals often encounter barriers to exercise such as obesity, congestive heart failure and atherosclerotic cardiovascular disease (ASCVD) itself. On top of unwillingness, these conditions make it often difficult to adhere to exercise^5, 6^. An alternative way to become physically active is eccentric exercise (EE), first described by researchers from the University of Bern, Switzerland^7, 8^.

At first glance, the concept of EE appears unusual. EE is defined as muscle work characterized by counteracting passive elongation of a muscle. An example is hiking downhill, where the muscle force reduces muscle elongation and thereby decelerates the hike. Eccentric exercise thus is the counterpart to concentric exercise which is characterized by active shortening of muscles as in hiking uphill. Mountains provide an excellent opportunity to apply the concept of both, concentric and eccentric exercise.

In pilot studies, effects of uphill and downhill hiking on glucose metabolism and blood lipids were demonstrated and the benefit of downhill hiking appears to depend on the energy expenditure^9^. Furthermore EE economically improves glucose tolerance and LDL cholesterol^10^. Even a small intervention such as descending stair walking solely could show positive effects such as increasing muscle strength in elderly or obese participants^11, 12^. Therefore it is a promising exercise modality for individuals who are not able to participate in more strenuous exercise regimens^10^. However, there are still only a few and mostly small, studies focusing on eccentric exercise (EE)^13–15^, underlying the necessity of larger controlled clinical trials.

With the present large, controlled proof-of-concept study, we tested the hypothesis whether EE is able to improve insulin sensitivity, triglyceride handling, lower body weight and inflammation when compared to a sedentary control group.

## Methods

We developed a two-month protocol encompassing hiking downwards in the western Austrian Alps close to our research center. Hiking downwards represents predominantly eccentric exercise. Although it is not purely eccentric muscle work, this term is used throughout the manuscript to refer to this specific training type.

The study was approved by the ethics committee of the state of Vorarlberg, Austria. The study protocol conforms to the ethical guidelines of the 1975 Declaration of Helsinki. All participants gave written informed consent.

### Sample size calculation

The sample size calculation for the main hypothesis—improvement of the lipid profile with concentric muscle training—results in a sample size of n = 25 for a significant reduction in LDL cholesterol assuming a mean value of m = 136.5 mg dl^−1^ and a training-related decrease of 5% with a power of 90 and an alpha of 0.05. In order to enable subgroup analyzes and assuming a drop-out rate of approximately 10%, the planned sample size was 100 study participants.

### Study participants

Sedentary healthy men and women aged ≥ 20 years were invited by advertisement in the local printed media, as well by local TV spots. Exclusion criteria were: previous physical exercise ≥ 3 times per week with a duration of ≥ 30 min per exercise bout within the last four months; heavy smoking > 20 cigarettes day^−1^; regular alcohol consumption > 60 g day^−1^; established musculoskeletal disease; a history of cardiovascular disease; diabetes mellitus; unwillingness to stay in the area for the whole study period; regular intake of any medication. Subjects who agreed to participate in the protocol then were allocated randomly in a 5:1 fashion either to the active exercise group or to the control group.

**Figure 1:**
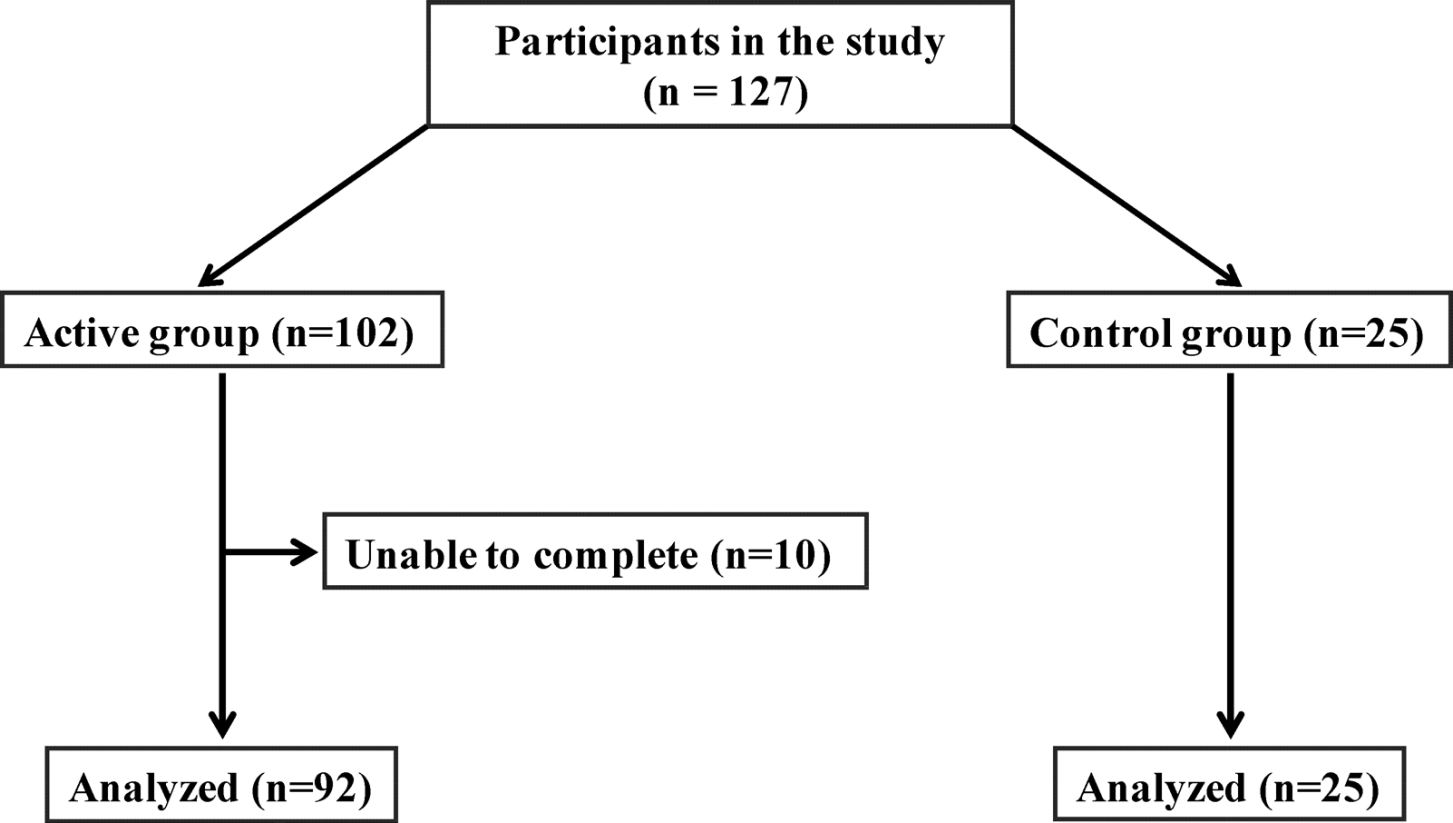
Flow chart of the study design.

The 127 volunteers (42 men and 85 women; mean age 49.0 ± 13.2 years) without any exclusion criterion were enrolled into the study (Fig. 1). All participants gave written informed consent. From the 127 individuals, 102 were allocated to the active group, and 25 to the control group. Of the 102 active group participants, 10 were unable to complete the strength program and 92 completed the study with a full data set.

### Study protocol

Participants belonged to one of two groups matched for age, gender, and smoking status: an active group (*n* = 102) performing two months of eccentric exercise, and a control group (*n* = 25) undertaking no additional sporting activities.

At baseline, the heart rate at the individual anaerobic threshold—HR_AT_; heart rate at a capillary lactate concentration of 4 mmol l^−1^^16^ was determined in the active group by a stepwise treadmill test. The target heart rate interval for the training program was set at − 25 to + 15 beats per minute around HR_AT_^9^. During the exercise bouts the heart rate was monitored by a portable pulse-meter with the aim the participants could adjust the hiking speed themselves.

The participants in the active group were instructed to follow the prescribed exercise regimen three to five times per week before the study was started. Our study protocol did not allow any other sporting activities during the study period. Thus, energy expenditure during everyday activities was kept constant at baseline level. To allow for real-life conditions, participants exercised on their own without an instructor. However, once a week, one of the authors was available at the training site for questions and advice.

The exercise comprised a steady downhill hike on a three-meter-wide mountain path over a distance of 2.9 km, and a difference in altitude of 540 m, thus the average slope was − 19%. As described in our pilot study, to estimate energy expenditure we used the approach published by Minetti et al.^9, 17, 18^. Hiking poles were used to involve the upper extremities in the exercise and also to protect joints. A cable car was used for the uphill route. Each participant was equipped with a personal pulse meter to monitor the heart rate during every exercise session. The probands were instructed to adhere to the pre-calculated heart rate range. Particular care was taken to ascertain adherence to the protocol. For that purpose, participants received complimentary tickets for the cable car. Every ride was recorded electronically, so that compliance was monitored.

All participants were advised to keep their nutrition constant. Dietary recall protocols were obtained from the active group, and calorie intake as well as dietary composition was recorded.

Metabolic testing was performed at baseline and after 8 weeks of eccentric exercise in the intervention group and after 8 weeks in the control group. Because we aimed at investigating the sustained and not the acute effects of exercise, testing was not performed during exercise, but started in the morning 36–40 h after the last exercise bout^9^. After a 12 h overnight fast, lipids and lipoproteins, insulin, creatine kinase (CK) activity and C-reactive protein (CRP) were measured, and a standardized 10-h oral lipid tolerance test was performed^19, 20^; before and 2, 4, 6, 8, and 10 h after a standardized test meal, serum triglyceride levels were determined. After another 12 h overnight fast, participants underwent a standardized 75-g oral glucose tolerance test (OGTT) on the morning of the following day^21^.

### Laboratory analyses

Biochemical analyses were performed using standard techniques, as described previously^22–24^. The serum levels of triglycerides, total cholesterol, low-density lipoprotein (LDL) cholesterol, and high-density lipoprotein (HDL) cholesterol were determined by using enzymatic hydrolysis and precipitation techniques (Triglycerides GPO-PAP, CHOD/PAP, QuantolipLDL, QuantolipHDL; Roche, Basel, Switzerland) on a Hitachi-Analyser 717 or 911. Glucose levels were measured enzymatically from venous fluoride plasma by the hexokinase method (Roche, Basel, Switzerland) on a Hitachi 717 or 911. Serum insulin was measured by an enzyme immunoassay on an AIA 1200 (Tosoh, Tokyo, Japan); as a measure of fasting insulin resistance, we used the homeostasis model assessment (HOMA-IR: (fasting serum insulin (μU ml^−1^) × fasting plasma glucose (mmol l^−1^)/22.5)), which yields reliable estimates of fasting insulin resistance^25^. Apolipoprotein B was measured on a Cobas Integra 800 (Roche, Basel, Switzerland).

### Statistical analysis

Descriptive statistics for continuous variables are given as means ± SD. Between-group differences were tested with Mann–Whitney *U* tests for continuous and with chi-squared tests for categorical variables; paired non-parametric Wilcoxon tests were used to test within-group changes over time for statistical significance; p-values < 0.05 were considered to indicate statistical significance. Results are given as mean ± standard deviation if not denoted otherwise. All analyses were performed with the software package SPSS 20.0 for windows.

## Results

At baseline, participants of the active and control groups had comparable body weight, body mass index, waist-to-hip ratio, and blood pressure values (Table 1). During the study period, 10 participants were unable to complete the training program: all due to lack of time. Thus, 117 patients (92 active group, 25 control group) completed the 8-week study period and were included in the analyses (Fig. 1).

**Table 1:** Baseline characteristics of the study participants.

	Active group	Control group	p-value
Characteristics n	92	25	
Age (years)	50 ± 10	48 ± 16	0.931
Male gender (%)	32.6	36.0	0.750
Waist circumference (cm)	103 ± 12	96 ± 16	0.037
Hip circumference (cm)	107 ± 11	104 ± 11	0.087
Waist to hip ratio	0.96 ± 0.08	0.92 ± 0.10	0.133
Smoking (%)	13.7	16.0	0.791
Systolic blood pressure (mmHg)	129 ± 16	129 ± 16	0.847
Diastolic blood pressure (mmHg)	87 ± 9	85 ± 10	0.246

Adherence to eccentric exercise in the intervention group was particularly good. The median frequency of walking per week was 3.2 ± 0.8 (interquartile range 2.9–3.9). Nobody ever exercised more than five times a week. The median exercise time was 40 min. The intake of energy, fat and carbohydrates did not change from baseline to the end of the study in the intervention group (7174 ± 2335 vs. 8055 ± 3402 kJ day^−1^; p = 0.358, 66.4 ± 27.6 vs. 76.9 ± 33.6 g d^−1^; p = 0.365, 193.0 ± 65.5 vs. 211.5 ± 108.4 g d^−1^; p = 0.926, respectively). No significant change was found within the control group.

**Figure 2:**
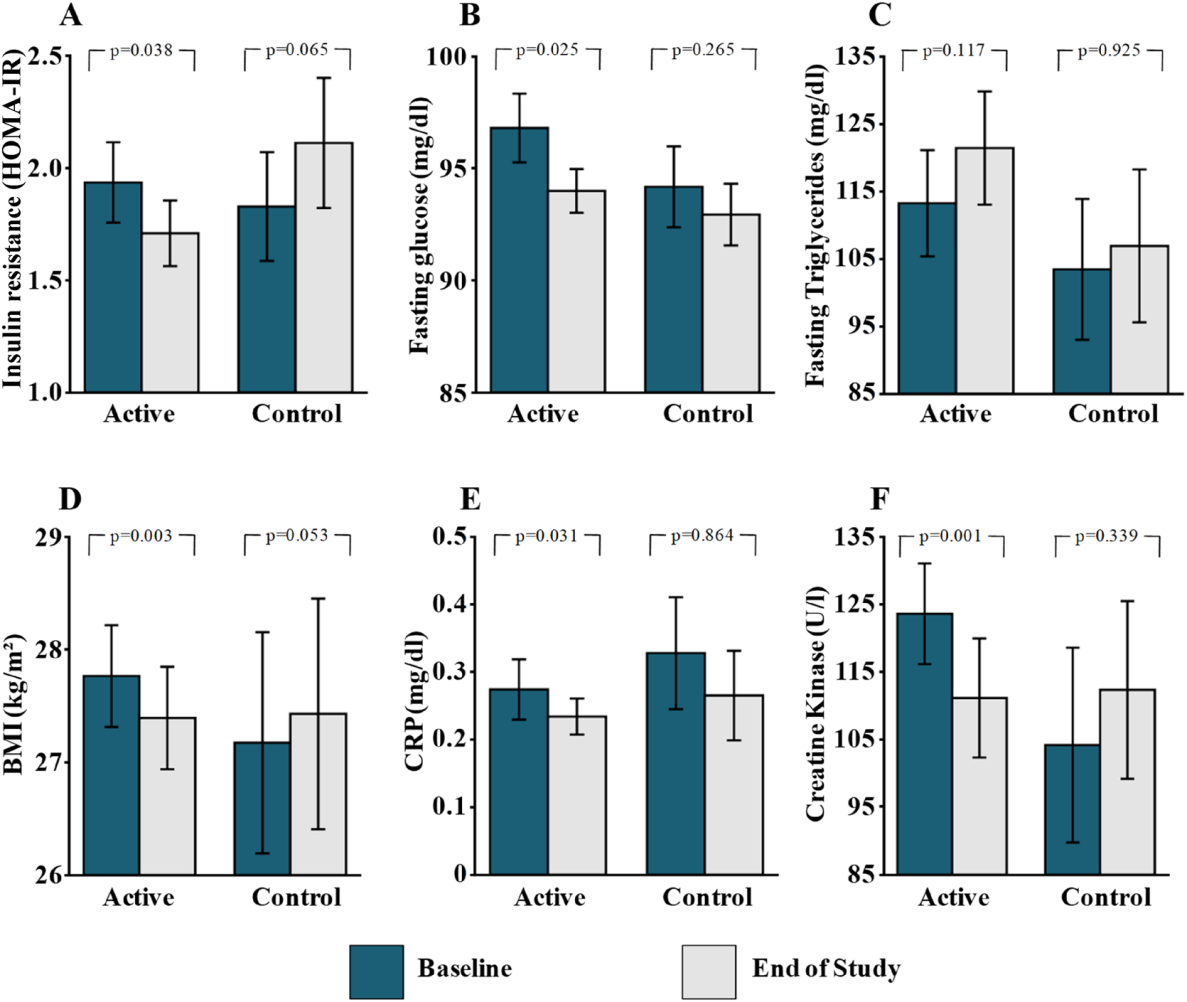
*BMI* Body mass index, *CRP* C-reactive protein, *CK* creatinine kinase. Mean changes in insulin resistance (**A**), fasting glucose (**B**), fasting triglycerides (**C**), BMI (**D**), CRP (**E**) and CK (**F**) with eccentric endurance exercise and in the control group. BMI denotes body mass index, insulin sensitivity is measured by the HOMA index calculated by the formula insulin (mU l^–1^) × fasting plasma glucose (mmol l^–1^)]/22.5. CK denotes creatine kinase.

Eccentric exercise induced improvements in insulin resistance (Fig. 2). HOMA-IR decreased from 1.94 ± 1.65 (µU^−1^ ml) × ((mmol/l)^−1^22.5) at baseline to 1.71 ± 1.36 (µU^−1^ ml) × ((mmol/l)^−1^22.5) (p = 0.038) in the active group, whereas it did not change significantly in the control group (1.83 ± 1.21 vs. 2.11 ± 1.45 (µU^−1^ ml) × ((mmol/l)^−1^22.5); p = 0.065). Fasting insulin tended to decrease in the active group (8.0 ± 6.4 vs. 7.2 ± 5.4 mU l^−1^; p = 0.056), whereas it increased in the control group (7.82 ± 5.27 vs. 9.17 ± 6.28 mU l^−1^; p = 0.035). Also, fasting glucose decreased in the interventional group after the 8-week training period, from 97 ± 15 to 94 ± 9 mg dl^−1^ (p = 0.025) but not in the control group (94 ± 9 vs. 93 ± 7 mg dl^−1^; p = 0.265).

Among fasting lipids, eccentric exercise did not improve total cholesterol, LDL-C or HDL-C in the active group. In contrast, Apo B significantly decreased both in the active (94 ± 28 vs. 90 ± 31 mg dl^−1^; p = 0.002) and in the control group (81 ± 20 vs. 78 ± 15 mg dl^−1^; p = 0.005). However, the LDL-C/ApoB ratio significantly increased in the intervention group (1.62 ± 0.16 vs. 1.65 ± 0.20; p = 0.003) but decreased in the control group (2.06 ± 1.95 vs. 1.73 ± 1.78; p = 0.045).

**Figure 3:**
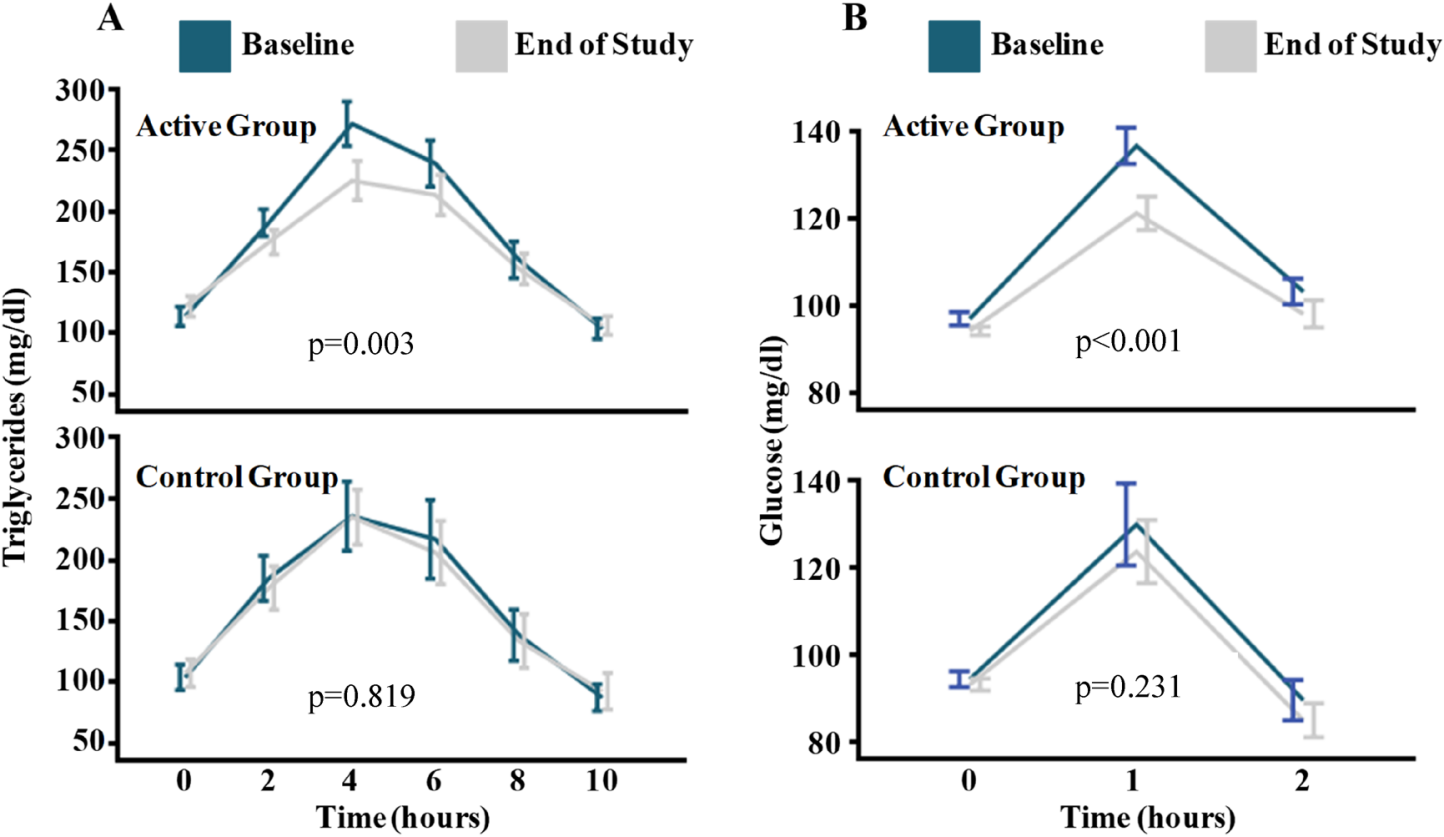
Comparison of the effects of eccentric endurance exercise with those of the control group on mean changes in postprandial triglyceride (**A**) glucose values (**B**).

Importantly, eccentric exercise training significantly improved postprandial metabolism (Fig. 3). Glucose tolerance improved in the eccentric exercise group (238 ± 50 vs. 217 ± 47 mg dl^−1^ h^−1^; p < 0.001) but not in the control group (221 ± 56 vs. 212 ± 43 mg dl^−1^ h^−1^; p = 0.231). Similarly, the intervention caused improvement in triglyceride tolerance (1923 ± 1295 vs. 1670 ± 1085 mg dl^−1^ h^−1^; p = 0.003), whereas triglyceride tolerance was not changed in the control group (1740 ± 976 vs. 1700 ± 955 mg dl^−1^ h^−1^; p = 0.819).

Even though no changes in diet were observed, BMI decreased in the eccentric exercise group during the 8-week intervention period (27.7 ± 4.3 vs. 27.4 ± 4.3 kg m^−2^; p = 0.003) but not in the control group (27.2 ± 4.9 vs. 27.4 ± 5.1 kg m^−2^; p = 0.053). Furthermore, CRP significantly decreased following eccentric exercise training (0.27 ± 0.42 vs. 0.23 ± 0.25 mg dl^−1^; p = 0.031) but did not change significantly in the control group (0.33 ± 0.41 vs. 0.26 ± 0.33 mg dl^−1^; p = 0.864). Finally, CK, a marker of muscle injury, was significantly decreased by eccentric exercise (125 ± 71 vs. 111 ± 84 U l^−1^; p = 0.001) but did not change significantly in the control group (104 ± 72 vs. 112 ± 66 U l^−1^; p = 0.339).

No skeletal or joint problems were reported, and no accidents occurred.

## Discussion

In this controlled study we describe multiple beneficial effects of EE. Not only the broad array of effects but also their magnitude is unexpected because EE is less strenuous than usual mixed or pure concentric exercise. We observed significant positive effects on insulin sensitivity and fasting and post-challenge glucose levels. Furthermore, we found a pronounced improvement of post-challenge triglycerides. Moreover, the inflammatory marker CRP decreased by the intervention. Finally, weight loss was achieved. Many these results are novel and have not previously been demonstrated by a controlled large study of EE.

HOMA-IR, reflecting fasting insulin resistance, improved after eight weeks of eccentric exercise training in line with lower fasting glucose levels. Also, glucose tolerance was improved, with a reduction of the area under the glucose-time curve by 9%. This is a strong effect considering the non-diabetic state of our participants at baseline^26, 27^. The clinical relevance of this finding is obvious as impaired glucose tolerance has been demonstrated to be a significant risk factor for coronary atherosclerosis and all cause mortality^28–30^. Muscle damage due to a single bout of downhill running caused an increase of insulin sensitivity^31^. In good accordance, our data show that sustained and repeated exercise has a similar effect. Consistently elevated vastatin concentrations in patients with T1DM can be lowered by regular physical exercise^32^.

Furthermore, eccentric exercise training exerted pronounced effects on postprandial triglyceride excursions (triglyceride tolerance)^20^. The area under the curve over 10 h after the standardized fat challenge decreased by 13%. This effect might be due to bodyweight loss, eccentric exercising has been reported to exert a weight-independent effect on triglyceride tolerance occurring even after one bout^33^. Another factor could be the association with muscle damage, but this effect decreases in further bouts^34^. As the extent of postprandial triglyceridemia consistently correlates with coronary artery disease and inflammation^35–37^, reduction exerted by eccentric exercise should be of prognostic relevance.

Our protocol of eccentric exercise, however, did not affect several other metabolic parameters: HDL-C did not improve, which may be due to its quite high baseline level and to the relatively short duration of the intervention. To raise HDL-C levels, interventions lasting at least half a year are necessary^38–40^.

Moreover, LDL-C was not improved significantly. Because LDL-C is not a typical feature of the insulin resistance syndrome, this finding is not surprising. However, the LDL-C/Apo B ratio was increased significantly by our protocol, pointing to an increase of LDL particle size which is generally seen when fasting and particularly postprandial triglyceridemia is reduced; this increase is considered antiatherogenic^39, 41^.

Although our findings emerged from a real-life study, no definite pathophysiological conclusions regarding metabolic mechanisms can be derived, it is tempting to consider which basic myocellular mechanisms could be behind the observed effects. The changes found between the baseline and the 8-week results appear causal by the exercise intervention: All possible confounders were unchanged between baseline and the 8-week examination, e.g. dietary habits or other forms of activity. The only change brought about was eccentric exercise.

Improvement of insulin sensitivity certainly is the cornerstone for the findings obtained on glucose and lipid metabolism as well as on reduced markers of inflammation. It is now well established that muscle tissue secretes regulatory cytokines and peptides, called myokines, and skeletal muscle can be viewed as an endocrine and paracrine organ^42, 43^. Some of the myokines are of high interest for metabolic research in exercise, one of them, meteorin-like 1, is released in response to downhill running in mice^44^. The view of the myocyte as an endocrine cell resembles that of the adipocyte and adipokines. It will be an important future part of research to investigate myokine adaptations in response to EE.

Safety and tolerability of our protocol were excellent and side effects were minimal. Muscle soreness is usually a problem with EE, but only for the first 1–3 bouts, as has been reported earlier in a pilot study^9^. Indeed, our patients did not report any muscle soreness during the investigational period. This is underlined by the objective finding of not only unincreased but rather slightly and significantly decreased serum activities of creatine phosphokinase^45^.

Also it has been demonstrated that eccentric exercise induced adaptations in muscle are not a result of inflammation^46^. Consistently our data even demonstrate that our eccentric exercise regimen led to decrease of the inflammation marker CRP.

Open questions remain as to the practical opportunities arising from these data. The classical type of EE, as in our study, is hiking downhill in the mountains. However, some alternatives are possible: in the initial program at the University of Bern, a special bicycle with braking facilities was constructed. Similar applications are feasible at fitness centers. Others use stairs^12^, a SmartEscalator device^11^, or knee extensors for leisure EE^15, 46^, to name a few.

Described positive effects reached by EE regarding lipid- and glucose metabolism, as well as inflammation are often the primary target of expensive and resource consuming drug treatments. Recent randomized controlled trials (RCTs) now provide evidence that drug treatment to decrease serum triglycerides by eicosapentaenoic acid^47^ or to lower low-grade inflammation by canakinumab^48^ or colchicine^49^ significantly and pronouncedly reduce cardiovascular events ASCVD. More trials on triglyceride lowering are currently underway. Also, pioglitazone-induced reduction of insulin resistance has been a key to a better outcome^50^. Although beneficially in secondary prevention, such drug therapy with its costs and inherent toxicity is not the first choice in primary prevention. Therefore, non-pharmacological intervention for these targets is of utmost academic and public interest in primary prevention^51^.

This study has several strengths and some limitations. As strengths we regard (i) the controlled design; (ii) the relatively large number of participants undergoing this type of intervention; (iii) the real-life conditions; (iv) the exact recording of compliance by the cable car tickets; (v) the schedule of metabolic testing apart from acute exercise bouts, (vi), the fact that insulin resistance and its sequelae were significantly improved in non-diabetic subjects, and, (vii) a small “research-to-practice gap”^4^. One limitation inherent in the study design may be the relatively short duration of the protocol. However, it was long enough to ascertain the significance of the findings and the case number calculation considered the duration of intervention.

In a pilot cross-over study comparing concentric and eccentric exercise we could find that the most pronounced effect of concentric exercise is lowering lipids, but not glycaemia. In terms of metabolic tolerance, eccentric exercise improved glucose tolerance, whereas concentric exercise improved triglyceride tolerance. Based on those data the present protocol focused on eccentric exercise vs. sedentary control and not versus concentric exercise, which might be considered as a limitation of the present study^9^.

Similarly in a previous study it was reported that energy expenditure was 72% less in eccentric than concentric exercise (442 kcal per concentric exercise bout v.s. 127 kcal eccentric exercise bout)^10^. Such a comparison was irrelevant against the sedentary controls in our protocol.

Our data on the decrease of BMI are important and highly significant (p = 0.003). In addition, it would be interesting to study body composition and body-fat in further studies.

In conclusion, we provide promising results of this controlled study on EE. Insulin sensitivity, glucose and lipid metabolism, and markers of inflammation, as well as body weight, are significantly improved by a simple and practically feasible exercise regimen. At a time when lowering triglycerides and inflammatory markers gain renewed therapeutic interest, these data are encouraging for primary and secondary prevention of ASCVD.

## Data Availability

The datasets generated during the current study are available in the VIVIT-Institute repository and from the corresponding author on reasonable request.
